# Associations Between Toxic Metal Exposure and Childhood Nephrotic Syndrome

**DOI:** 10.1016/j.ekir.2026.106357

**Published:** 2026-02-12

**Authors:** Cal Robinson, Nowrin Aman, Harshita Arora, Tonny H.M. Banh, Josefina Brooke, Vaneet Dhillon, Mackenzie Garner, Tanay Joshi, Christoph Licht, Ashlene McKay, Sruti Prabakaran, Rachel Pearl, Seetha Radhakrishnan, Nithiakishna Selvathesan, Chia Wei Teoh, Jovanka Z. Vasilevska-Ristovska, Rulan S. Parekh

**Affiliations:** 1 Department of Paediatrics, Division of Nephrology, The Hospital for Sick Children, Toronto, Ontario, Canada; 2 Child Health Evaluative Sciences, Research Institute, The Hospital for Sick Children, Toronto, Ontario, Canada; 3 Department of Medicine, Women’s College Hospital, Toronto Ontario, Canada; 4 School of Medicine, Royal College of Surgeons in Ireland University of Medicine and Health Sciences, Dublin, Ireland; 5 Department of Pediatrics, William Osler Health Systems, Brampton, Ontario, Canada

**Keywords:** children, heavy metal, mercury, minimal change disease, nephrotic syndrome, toxic metal

## Abstract

**Introduction:**

Mercury intoxication causes nephrotic syndrome in children and adults. It is unknown if chronic low-grade metal exposure is a risk factor for childhood nephrotic syndrome. We aimed to evaluate the associations between metal exposure and childhood nephrotic syndrome outcomes.

**Methods:**

We analyzed data from the Insight into Nephrotic Syndrome: Investigating Genes, Health, and Therapeutics (INSIGHT) study, a prospective childhood nephrotic syndrome cohort. We included children (aged 1–18 years) with nephrotic syndrome from the Greater Toronto Area, Canada, excluding congenital or secondary nephrotic syndrome. Toenail clippings were collected and tested for total mercury, copper, arsenic, selenium, cadmium, tin, and lead concentrations. We evaluated associations between individual and mixed metal concentrations and relapse rate, steroid resistance, frequent relapses or steroid dependence, and steroid-sparing immunosuppression use using regression analysis.

**Results:**

Nail samples from 298 children with nephrotic syndrome were analyzed. Less than 1% had nail mercury and 10% had other metal concentrations above the reference limits. None had metal concentrations in a potentially toxic range. There were no significant associations between nail mercury concentration and nephrotic syndrome relapses (relative rate: 1.05, 95% confidence interval [CI]: 0.96–1.14), steroid resistance (odds ratio: 0.90, 95% CI: 0.59–1.32), frequent relapses or steroid dependence (odds ratio: 1.01, 95% CI 0.83–1.24), or steroid-sparing medication use (odds ratio: 1.12, 95% CI: 0.92–1.35). In addition, concentrations of other individual or combined metals were not associated with nephrotic syndrome outcomes.

**Conclusion:**

Evidence of high exposure to mercury and other toxic heavy metals is rare among Canadian children with nephrotic syndrome. We did not find evidence that chronic exposure to toxic heavy metals is associated with nephrotic syndrome outcomes.

## Graphical abstract

**Figure:**
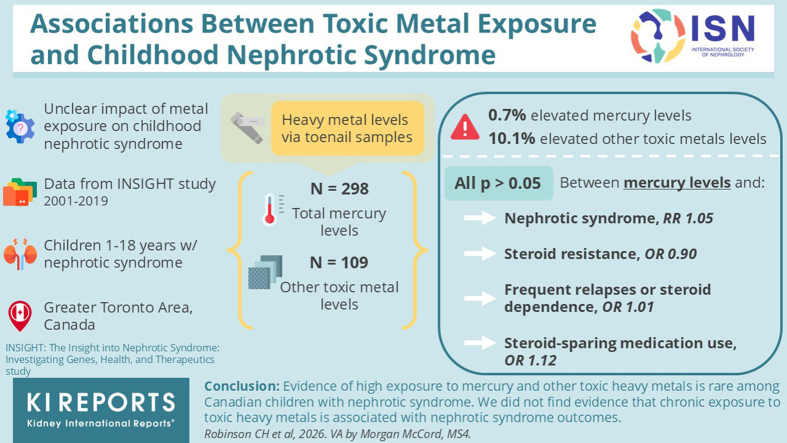

Toxic heavy metals are metallic elements, such as mercury, arsenic, cadmium, and lead with known adverse effects on human health.^1^^,^^2^ In developed countries, pollution of heavy metals is largely due to historical emissions from industrial applications (e.g., mining, smelting, and battery production), which persist in the environment long after emissions have ceased.^3^ Contemporary sources of heavy metal pollution include agricultural pesticides and fertilizers (arsenic and cadmium), dental amalgam and predatory fish consumption (mercury), traditional medicines (mercury and lead), and paint (lead).^1^ Children are particularly susceptible to environmental heavy metal exposure, because they are at a higher risk of accidental ingestion from hand-to-mouth activity and they inhale more air, drink more water, and consume more fluid by body surface area than adults.^2^ Heavy metals can accumulate in the kidney, resulting in toxicity.^1^ Acute toxicity from high-level exposures can cause acute kidney injury and proximal tubular dysfunction.^1^ However, the effects of chronic low-grade toxic metal exposures are not consistently associated with kidney disease. Occupational exposure to arsenic, cadmium, and lead have been associated with chronic tubulointerstitial damage and progressive chronic kidney disease in adults, although studies are conflicting.^2^^,^^4^, ^5^, ^6^

Acute mercury exposure is associated with nephrotic syndrome, whereas chronic exposure is not a known risk factor. Mercury exists in the following 3 distinct forms: (i) elemental mercury (e.g., industrial uses, dental amalgams, mercury thermometers), (ii) inorganic mercury (e.g., mining, medicinal products such as skin lightening creams, traditional herbal medicines, and historical teething powders), and (iii) organic mercury (e.g., aquatic food chain).^2^ High-grade exposure to mercury-containing products such as skin lightening creams and traditional herbal medicines can cause nephrotic syndrome in children and adults.^7^, ^8^, ^9^, ^10^, ^11^ Although membranous nephropathy is the classical histopathological finding, minimal change disease is reported in up to two-thirds of cases.^7^^,^^11^, ^12^, ^13^ In animal models, mercury has been shown to stimulate the immune system and result in glomerular immune complex deposition.^14^, ^15^, ^16^, ^17^ Idiopathic nephrotic syndrome is one of the most common childhood kidney diseases, and the pathophysiology remains poorly understood.^18^ Data are lacking on potential associations between chronic exposures to low-grade mercury and other toxic metals and childhood nephrotic syndrome. This is a key knowledge gap, because understanding the immune-environment interactions of nephrotic syndrome is necessary to identify therapeutic targets and develop preventative care strategies. Therefore, our aim was to assess for evidence of toxic heavy metal exposure among children diagnosed with nephrotic syndrome and to evaluate associations between heavy metal exposure, nephrotic syndrome disease activity, and immunosuppressive treatment response.

## Methods

### Study Design and Population

The INSIGHT study (ClinicalTrials.gov identifier NCT1605266)^19^ is a prospective observational cohort study including children (aged 6 months–18 years) diagnosed with childhood nephrotic syndrome after 1996. Nephrotic syndrome was defined by standardized Kidney Disease Improving Global Outcomes criteria.^20^ Detailed baseline sociodemographic and clinical data were collected for INSIGHT participants from diagnosis. Participants underwent annual follow-up until clinic discharge, typically 5 years after their last relapse or at the age of 18 years. INSIGHT participants were treated at the Hospital for Sick Children, McMaster Children’s Hospital, or community hospitals in the Greater Toronto Area. The study received research ethics approvals at each clinical center. We included all children diagnosed with nephrotic syndrome from 2001 to 2019 and enrolled in INSIGHT before March 2020 that had toenail samples collected at any study visit. Children were excluded if they had monogenic nephrotic syndrome, secondary nephrotic syndrome (e.g., lupus nephritis), or biopsy-proven membranous nephropathy or membranoproliferative glomerulonephritis.

### Research Ethics and Consent

Research ethics approval for INSIGHT was obtained from The Hospital for Sick Children, William Osler Health System (Brampton Civic Hospital and Peel Memorial Centre), Scarborough Health Network (Centenary Hospital) and McMaster Children's Hospital. Informed consent for the study was obtained from all participants.

### Toenail Samples

Toenail clippings were collected from INSIGHT participants at a single study visit (typically the enrollment visit) between March 2011 and June 2024. Toenail clippings collection for environmental metal testing was a prespecified but optional component of INSIGHT from study initiation.^19^ At least 3 clippings from different toenails (without nail polish or acrylic) were collected with single-use chromium-free nail cutters. Toenail clippings were stored in a sealed envelope within a sealed plastic bag to prevent contamination. Toenail collection after March 2020 was limited because of COVID-19–related disruptions to in-person clinic visits and equipment shortages. Nails are a more reliable biological sample to measure chronic toxic metal exposure than blood or urine.^21^, ^22^, ^23^, ^24^ Because toenails typically grow at a rate < 1 mm/wk, clippings are an indicator of toxic metal exposures over the prior 6 to 12 months.^24^^,^^25^ Toenail samples were used because they are easy and painless to collect, and are less likely to be contaminated than fingernail or hair samples.^26^

### Toxic Metal Analysis

All analyses were conducted in the Western University Biotron laboratory, which is accredited by the Canadian Association for Laboratory Accreditation and adheres to ISO/IEC 17025 standards. Toenail samples were tested for total mercury concentration by thermal decomposition, amalgamation, and atomic absorption using the Milestone Direct Mercury Analysis-80 evo system, according to the US Environmental Protection Agency method 7473.^27^ Toenail concentrations of other toxic metals (copper, arsenic, selenium, cadmium, tin, and lead) were analyzed by inductively coupled plasma mass spectrometry (ICP-MS) using the Agilent 7700x system, according to modified US Environmental Protection Agency 200.8 and 3051A methods.^28^^,^^29^ For the ICP-MS analysis, samples were digested using 2 ml of concentrated aqua regia and then diluted to a volume of 50 ml for analysis. The sample weight used for digestion varied by toenail ranging from 1.4 to 90 mg. Internal laboratory quality control (QC) measures were conducted (Supplementary Table S1) and 37 (11%) paired replicates were sent for further validation. Laboratory personnel were blinded to these additional QC samples. Toxic metal concentrations were reported in μg/g of nail sample. Published normative reference and toxicity thresholds are described in Supplementary Table S2. We contacted all participants with high nail metal concentrations to evaluate for symptoms of toxicity, dietary habits, and to conduct confirmatory blood and urine testing.

### Disease Outcomes

We evaluated the association between individual and combined toxic metal nail concentrations and longitudinal nephrotic syndrome outcomes. Our primary outcome of interest was relapse rate (number of relapses/patient-year) throughout follow-up. Secondary outcomes were classification with steroid-resistant nephrotic syndrome (defined as failure to achieve complete remission after 8 weeks of standard steroid therapy), classification with frequently relapsing or steroid-dependent nephrotic syndrome (by 1 year after diagnosis among steroid-sensitive participants), and steroid-sparing immunosuppressive medication use at any time during follow-up (e.g., cyclophosphamide, calcineurin inhibitors, mycophenolate, or rituximab). Nephrotic syndrome classification was based on the 2021 Kidney Disease: Improving Global Outcomes clinical practice guidelines.^30^

### Statistical Analysis

All statistical analyses were conducted using R Statistical Software (version 4.4.0, Vienna, Austria) and a *P*-value < 0.05 was considered significant. We reported baseline participant characteristics among the overall cohort using descriptive statistics. For each toxic metal, we determined the number (%) of participants with levels below the method detection limit (MDL), detectable, and above reference limits. Among participants with samples above the MDL, we calculated the mean and SD concentrations for each toxic metal. We evaluated the association between individual and combined toxic metal nail concentrations and relapse rate throughout follow-up period using unadjusted negative binomial regression models, to address overdispersed count data. Nail metal concentrations were log-transformed for regression analyses to address right-skewed distributions. For samples with toxic metal concentrations below the MDL, we imputed these values using the MDL (i.e., the highest possible concentration for each sample). Missing data were not imputed for any covariates or outcomes. The associations between log-transformed nail metal concentrations and steroid resistance, frequent relapses or steroid dependence, and steroid-sparing medication use were evaluated using unadjusted logistic regression models. We assessed interactions between nail mercury concentration and ethnicity for these outcomes using interaction terms. As a sensitivity analysis, we evaluated the associations between log-transformed nail metal concentrations and each outcome after adjustment for age at diagnosis, biologic sex, and ethnicity as theoretical confounders.

To evaluate the combined effect of multiple toxic metal exposures on nephrotic syndrome outcomes, we conducted weighted quantile sum regression.^31^^,^^32^ These models calculate a weighted percentile index representing the overall weight of all metals and separate weights for each metal to determine their contribution to the weighted quantile sum index. Weighted quantile sum analyses were conducted using untransformed nail metal concentrations; 40% test, and 60% validation datasets, 100 bootstrap procedures, and 100 replicate retention validations were used to estimate weights and regression coefficients (linear regression for relapse rate and logistic regression for other outcomes).

## Results

### Participant Characteristics

Of 584 children with nephrotic syndrome enrolled in the INSIGHT study before March 2020, 298 (51%) had toenail samples collected at a median of 2.1 (interquartile range [IQR]: 0.6–4.5) years after diagnosis (Table 1). Among these 298 participants, the median age at nephrotic syndrome diagnosis was 4 (IQR: 3–6) years, 186 (63%) were males, 104 (36%) were South Asian, 87 (29%) were classified as frequently relapsing or steroid-dependent by 1 year after diagnosis, and 17 (6%) were classified as steroid-resistant during initial treatment. The characteristics of INSIGHT participants with and without nail samples collected were similar (Table 1). Median follow-up from nephrotic syndrome diagnosis was 7.0 (IQR: 4.0–10.1) years.

**Table 1:** Baseline characteristics of 757 children with nephrotic syndrome enrolled in INSIGHT, stratified by nail sample collection

Baseline characteristics	Children with nail samples (*n* = 298)	Children without nail samples (*n* = 459)	SMD
Age at diagnosis in yrs, median (IQR)	4 (3–6)	4 (3–6)	0.03
Sex, *n* (%)			0.03
Male	186 (63%)		
Female	108 (37%)	168 (37%)	
Ethnicity, *n* (%)			0.11
European	77 (26%)	105 (25%)	
South Asian	104 (36%)	138 (33%)	
East Asian	27 (9%)	50 (12%)	
Other	84 (29%)	132 (31%)	
Immigrant to Canada, *n* (%)	46 (16%)	94 (21%)	0.13
Rural residence, *n* (%)	10 (3%)	26 (6%)	0.12
Total family income, *n* (%)			0.75
< $50,000	82 (28%)	73 (16%)	
$50,000–$99,999	74 (25%)	78 (17%)	
≥ $100,000	93 (32%)	89 (19%)	
Missing/declined	45 (15%)	219 (48%)	
Household smoker, *n* (%)	24 (8%)	46 (10%)	0.07
Nephrotic syndrome classification^a^ by 1 yr after diagnosis, n (%)			
Never relapse	42 (14%)	52 (11%)	0.10
Infrequent relapses	148 (50%)	240 (52%)	
Frequently relapsing	20 (7%)	32 (7%)	
Steroid dependent	67 (23%)	111 (24%)	
Steroid resistant	17 (6%)	24 (5%)	
Kidney biopsy histopathology, n (%)			
Never biopsied	169 (58%)	286 (62%)	0.11
Minimal change disease	76 (26%)	111 (24%)	
Focal segmental glomerulosclerosis	31 (11%)	41 (9%)	
Other^b^	18 (6%)	21 (5%)	

IQR, interquartile range; SMD, standardized mean difference.

A difference > 0.1 is considered substantial.

a Nephrotic syndrome classification based on the 2021 Kidney Disease: Improving Global Outcomes (KDIGO) criteria.

b Other histopathological diagnoses included mesangial hypercellularity, diffuse podocytopathy not otherwise specified, acute tubular necrosis, and global glomerulosclerosis.

### Toxic Metal Exposure

**Figure 1:**
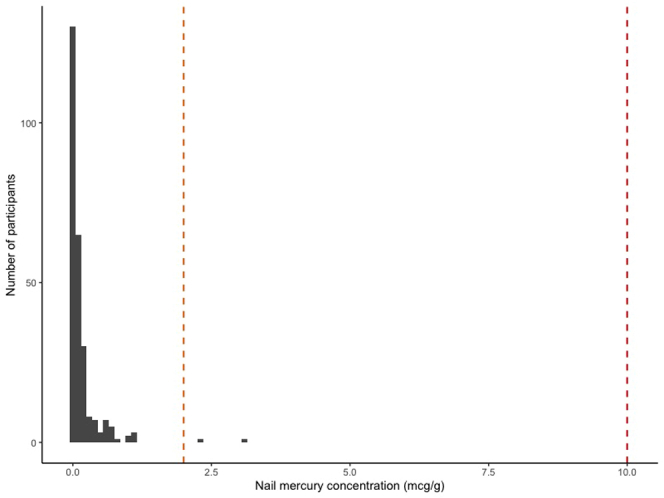
Distribution of nail mercury concentrations among 298 children with nephrotic syndrome. This figure shows the distribution of participant nail mercury concentrations in μg/g. The orange dashed line represents the upper limit of normal nail mercury concentration (2 μg/g of nail sample) based on World Health Organization guidelines. The red dashed line represents the lower toxicity limit (10 μg/g of nail sample).

Total mercury concentration was determined in all 298 nail samples (Table 2). After conducting this analysis, 109 participants (37%) had sufficient samples remaining to test other toxic metals by ICP-MS. Participants with sufficient samples for ICP-MS were older at sample collection (median age: 10.0 vs. 6.9 years) but were otherwise similar to those without sufficient samples. All internal laboratory QC standards were met (Supplementary Table S1) and MDLs for each toxic metal are reported in Table 2. Nail metal concentrations from blinded QC samples were largely concordant, 47 (52%) of pairs were both below the MDL and small absolute differences existed when both pairs had detectable levels. Overall, < 5 children (0.7%) had nail total mercury concentration above reference limits (Figure 1). Eleven of 109 children (10.1%) had a nail concentration of at least one other toxic metal (e.g., copper, tin, or lead) above reference limits (Supplementary Figure S1). However, no child had a nail concentration of mercury or another toxic metal in a suspected toxic range (Supplementary Table S2 and Supplementary Figure S1). Among the 13 children with any nail metal concentration above reference limits, the median age at diagnosis was 5.2 (IQR: 2.8–6.0) years, median sample collection was at the age of 1.1 (IQR: 0.8–4.9) years after diagnosis, 9 were male, 5 were of South Asian ethnicity, and 11 had infrequent or no relapses. Nine of these children were able to be contacted at median age of 10.5 (IQR: 9.3–12.0) years after nail sample collection. None reported any symptoms of metal toxicity or unusual dietary habits. Confirmatory blood and urine testing for toxic metals were conducted in 3 participants, which were normal.

**Table 2:** Toxic metal concentrations in nail samples from 298 children with nephrotic syndrome

Toxic metal	Nail concentration above reference limit^a^, *n* (%)	Below method detection limit^b^, *n* (%)	Above method detection limit^b^, *n* (%)	Mean (SD) concentration^c^, μg/g
Mercury (Hg), *n* = 298	< 5 (< 1%)	35 (11.8%)	263 (88.3%)	0.15 (0.31)
Copper (Cu), *n* = 109	5 (4.6%)	7 (6.4%)	102 (93.6%)	7.41 (5.69)
Arsenic (As), *n* = 109	0 (0%)	10 (9.2%)	99 (90.8%)	0.48 (0.31)
Selenium (Se), *n* = 109	0 (0%)	56 (51.4%)	53 (48.6%)	1.09 (0.35)
Cadmium (Cd), *n* = 109	0 (0%)	107 (98.2%)	< 5 (< 4%)	0.08 (0.08)
Tin (Sn), *n* = 109	< 5 (< 4%)	81 (74.3%)	28 (25.7%)	1.68 (1.85)
Lead (Pb), *n* = 109	< 5 (< 4%)	91 (83.5%)	18 (16.5%)	0.48 (1.85)

a Upper limit thresholds for nail toxic metal concentrations were as follows: mercury > 2 μg/g, copper > 20 μg/g, arsenic > 1 μg/g, selenium > 3 μg/g, cadmium > 0.2 μg/g, tin > 3.8 μg/g, and lead > 4 μg/g. All metal concentrations are reported in μg/g of nail sample. Cell sizes with < 5 participants are reported as < 5 for patient privacy.

b The method detection limits for nail toxic metals were as follows: mercury 0.07 ng, copper 0.18 μg/l, arsenic 0.08 μg/l, selenium 0.12 μg/l, cadmium 0.04 μg/l, tin 0.13 μg/l, and lead 0.2 μg/l.

c Mean (SD) toxic metal concentrations calculated among samples above the method detection limit. These values are not log-transformed.

### Association With Nephrotic Syndrome Outcomes

**Figure 2:**
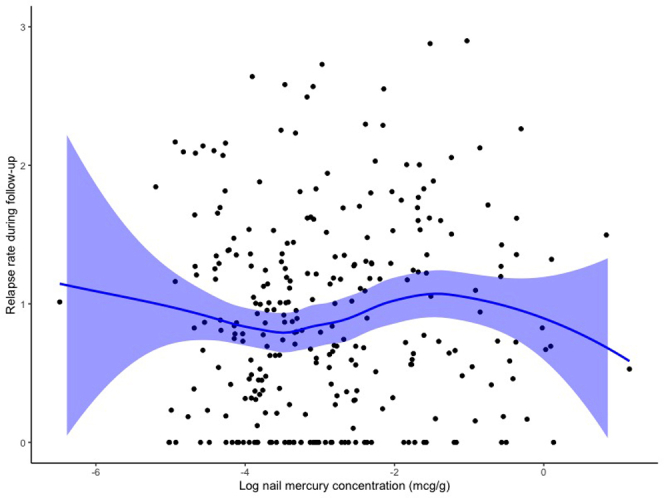
Correlation between log-transformed nail mercury concentration and relapse rate during follow-up among 298 children with nephrotic syndrome. The solid blue line represents a smoothed local regression line and blue shaded area represents its 95% confidence interval. There is no evidence of a significant correlation between nail mercury concentration and relapse rate (number of relapses/yr) throughout follow-up.

**Figure 3:**
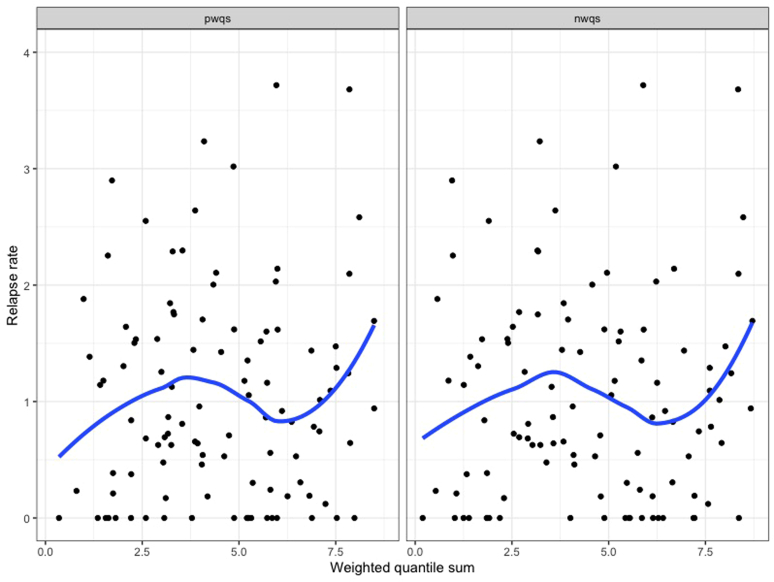
Correlations between mixed metal concentrations and relapse rate during follow-up among 298 children with nephrotic syndrome. The left panel of Figure 3 shows the pwqs regression for the association between the weighted sum of toxic metal concentrations (mercury, copper, arsenic, selenium, cadmium, tin, and lead) and relapse rate (number of relapses/yr) throughout follow-up. The right panel shows the nwqs regression results. There is no evidence of significant correlations between positive or negative weighted quantile sums of multiple toxic metal concentrations and relapse rate throughout follow-up. nwqs, negative weighted quantile sum; pwqs, positive weighted quantile sum.

There were no significant associations between nail concentrations of any individual toxic metal and nephrotic syndrome relapse rate throughout follow-up (Table 3). For mercury, its log-transformed nail concentration (in μg/g) was associated with a relative rate of 1.05 (95% CI: 0.96–1.14, *P* = 0.97) for relapses throughout follow-up (Figure 2). There were no significant associations between nail concentrations of any individual toxic metal and steroid resistance, frequent relapses or steroid dependence, or steroid-sparing immunosuppressive medication use (Table 3). There were no significant associations between the combined effect of multiple metal exposures (by weighted quantile sums) and relapse rate, frequent relapses or steroid dependence, or steroid-sparing immunosuppressive medication use (Supplementary Table S3 and Figure 3). There were no significant associations between any nail metal concentration and outcomes after adjusting for age, sex, and ethnicity (Supplementary Table S4). Further, we did not find evidence of a significant interaction between ethnicity and nail mercury concentration for any outcomes (all *P* > 0.05).

**Table 3:** Associations between toxic metal concentrations and childhood nephrotic syndrome outcomes

Exposure^a^	Relapse rate during follow-up^b^	Initial SRNS	FR- or SDNS by 1-year after diagnosis	Steroid-sparing medication use during follow-up
	RR (95% CI)^c^	OR (95% CI)^d^	OR (95% CI)^d^	OR (95% CI)^d^
Log mercury concentration (μg/g)	1.05 (0.96–1.14)	0.90 (0.59–1.32)	1.01 (0.83–1.24)	1.12 (0.92–1.35)
Log copper concentration (μg/g)	1.19 (0.86–1.62)	0.05 (0.00–0.97)	0.88 (0.40–1.84)	1.44 (0.70–3.15)
Log arsenic concentration (μg/g)	0.94 (0.75–1.19)	0.39 (0.08–1.65)	0.99 (0.58–1.71)	0.88 (0.52–1.46)
Log selenium concentration (μg/g)	1.08 (0.74–1.59)	0.56 (0.04–6.27)	1.32 (0.56–3.16)	0.89 (0.38–2.07)
Log cadmium concentration (μg/g)	0.97 (0.78–1.21)	0.51 (0.15–1.87)	1.00 (0.61–1.67)	0.95 (0.58–1.52)
Log tin concentration (μg/g)	0.97 (0.77–1.23)	0.27 (0.03–1.29)	0.91 (0.53–1.55)	0.93 (0.56–1.55)
Log lead concentration (μg/g)	0.96 (0.77–1.22)	0.40 (0.10–1.51)	0.74 (0.44–1.25)	0.97 (0.59–1.59)

CI, confidence interval; FR- or SDNS, frequently-relapsing or steroid-dependent nephrotic syndrome; OR, odds ratio; RR, relative rate; SRNS, steroid-resistant nephrotic syndrome.

All *P*-values > 0.05.

a Toxic metal concentrations were log-transformed. All metal concentrations are reported in mcg/g of nail sample.

b Relapse rate calculated as the total number of relapses/yr of follow-up time.

c Unadjusted relative rates were calculated using negative binomial regression models, to account for over-dispersion.

d Unadjusted odds ratios were calculated using logistic regression models.

## Discussion

Caregivers of children with nephrotic syndrome often voice concerns about the potential association between chronic exposure to mercury or other toxic heavy metals and nephrotic syndrome. In a large prospective childhood nephrotic syndrome cohort from a high-income country, no children had evidence of heavy metal exposure in a suspected toxic range. Less than 1% had evidence of high mercury exposure and 10% had evidence of high exposure to another heavy metal (copper, tin, or lead), but all levels were below toxicity thresholds. Because normative data for nail metal concentrations is scarce and typically based on population distributions (i.e., 95th percentile among healthy individuals), it was expected that some results would be above the upper limit of normal. Further, there were no significant associations found between individual or combined nail metal concentrations and nephrotic syndrome relapses, steroid resistance, or use of steroid-sparing immunosuppressive medications. These findings suggest that among Canadian children living in modern environmental conditions, exposure to toxic heavy metals is not a significant risk factor for worse longitudinal nephrotic syndrome outcomes.

Mercury toxicity is associated with nephrotic syndrome occurrence in adults and children.^7^, ^8^, ^9^, ^10^^,^^12^^,^^33^ Although the mechanisms remain uncertain, studies report mercury accumulation in the kidney, which stimulates the immune system, resulting in immune complex deposition.^7^ Mercury decreases B and T cell numbers and promotes an autoimmune response.^16^^,^^17^ Mercury-induced nephrotic syndrome is typically reported after acute exposure to mercury-containing cosmetic products (e.g., skin-lightening creams) or traditional herbal medicines.^7^^,^^8^^,^^12^ In a cohort of 172 Chinese adults with mercury poisoning, 94% were due to cosmetic products or traditional medicines and 24% of patients developed nephrotic syndrome (51% membranous nephropathy, 37% minimal change disease, and 9% mesangial proliferative glomerulonephritis).^7^ Compared with adults with primary minimal change disease, those with mercury-induced nephrotic syndrome have similar clinical and pathological characteristics, but experience a lower relapse rate (0% vs. 47%).^34^

Compared with acute mercury toxicity, there are very limited data on the potential relationship between chronic low-grade mercury exposure and nephrotic syndrome.^7^^,^^35^ Environmental exposure to mercury can occur from dental amalgam (“silver fillings”), proximity to industrial activities (e.g., mining, manufacturing, and electrochemical processes), and consumption of fish high on the aquatic food chain (e.g., swordfish and tuna; due to accumulation of organic methylmercury). The US Environmental Protection Agency, US Food and Drug Administration, and Health Canada all report that there is no evidence of health risk from mercury-containing dental amalgam in the general population, although they recommend avoidance in patients with impaired kidney function because of potential accumulation and neurotoxicity. Two clinical trials randomizing children (*N* = 534 and *N* = 507) with dental caries to receive either amalgam or resin composite fillings found no differences in kidney function or tubular injury over 5 to 7 years follow-up.^36^^,^^37^ Microalbuminuria was more common among children receiving dental amalgam (16% vs. 10%, *P* = 0.03) in one trial, but not the other. No previous studies have evaluated the association between mercury exposure and childhood nephrotic syndrome. In our study, the prevalence of nail mercury concentration above reference limits was < 1%. Among 5319 participants included in the Canadian Health Measures Survey, 1.6% of children and pregnant women had high blood mercury levels.^38^ This suggests that children with nephrotic syndrome are not exposed to higher levels of mercury than the general Canadian population. Further, no children in our study had nail mercury levels in a suspected toxic range and mercury levels were not associated with nephrotic syndrome relapses or treatment response.

Chronic exposure to other toxic heavy metals can occur in occupational settings (e.g., industrial or mining facilities) or because of contaminated food and water from historical industrial emissions.^2^ These toxic metals can accumulate in the kidney and cause acute kidney injury and proximal tubular dysfunction.^1^^,^^2^ However, the relationship between chronic low-grade exposure to these toxic metals in the environment and glomerular diseases is poorly understood. We found that 10% of children with nephrotic syndrome had a level of ≥ 1 toxic metal above reference limits, but none of these levels were in a suspected toxic range. Reference ranges for nail concentrations of most toxic metals are not well-established. These are often based on normative distributions in samples of healthy participants (i.e., 95th percentile), instead of toxicological effects. Thus, it is expected that some healthy individuals will have levels above the upper limit of normal without toxicity. There was no association between the concentration of any of these toxic metals individually or in aggregate with longitudinal nephrotic syndrome outcomes.

The study has multiple strengths. We included a large childhood nephrotic syndrome cohort with a median follow-up of 7 years, allowing us to investigate the association between mercury and other toxic metal exposures and longitudinal outcomes. Testing for toxic metals was conducted at an accredited laboratory using methods approved by the US Environmental Protection Agency, with robust QC measures. Nails are a reliable specimen to measure chronic exposure to toxic metals based on their slow growth rate.^21^, ^22^, ^23^, ^24^ Nail clippings measure metal exposures 6 to 12 months prior to sample collection.^24^^,^^25^

This study has several limitations. Cross-sectional collection of nail clippings at a single time point for each participant is a significant limitation, because nail metal concentrations may fluctuate over time with changes in diet, residential address, or other environmental exposures. Nail clippings were collected within 6 months and 1 year of diagnosis in only 22% and 32% of participants, respectively. Therefore, measured nail metal concentrations may not accurately reflect exposure at the time of nephrotic syndrome diagnosis in more than two-thirds of participants. This limits our ability to exclude a causal relationship between toxic metal exposures and nephrotic syndrome pathogenesis. Only 51% of INSIGHT participants enrolled before 2020 had nail samples collected for analysis, which could introduce selection bias. However, the characteristics of these participants were mostly comparable with those without nail samples collected. One exception was self-reported total family income, which participants without nail samples collected more commonly declined to answer (48% vs. 15% with nail samples). Lack of trust, concerns about confidentiality, and cultural differences may contribute to both lower response rates to socioeconomic questions and toenail sample collection, which potentially limits the generalizability of our findings. Approximately half of nail clipping samples had some encrusted dirt present, which introduces random error and may bias measured concentrations of toxic metals. Nail clippings were not decontaminated before analysis. Although there was ethnic and socioeconomic diversity in our study population, these findings may not be generalizable to low-to-middle-income countries where industrial pollution from toxic heavy metals is ongoing and could contribute to worse outcomes. Further studies among children with nephrotic syndrome in these settings is warranted. Another limitation is the lack of data of the presence of dental amalgam fillings, which may contribute to mercury exposure. Finally, after conducting direct mercury analysis for total mercury concentration, only 109 participants (37%) had sufficient samples remaining for ICP-MS analysis for other toxic metals. Considering that mercury was our primary toxic metal of interest based on the previous literature and caregiver concerns, this was prioritized for analysis. However, this may introduce selection bias into our analyses of other toxic metals, because samples of smaller mass (e.g., from younger children) were less likely to be analyzed.

## Conclusion

In a cohort of Canadian children with nephrotic syndrome living in modern environmental conditions, < 1% had levels of mercury and 10% had levels of other heavy metals (copper, tin, and lead) above reference limits. None of these children had levels of heavy metals in a toxic range. We did not find evidence of any associations between heavy metal exposure and adverse longitudinal nephrotic syndrome outcomes.

## Disclosure

All authors declared no competing interests.

## Supplementary Material

Supplementary File (PDF) **Supplementary File. Figure S1.** Nail toxic metal concentrations among 298 children with nephrotic syndrome. **Table S1.** Internal laboratory quality control measures of nail toxic metal concentration analysis. **Table S2.** Upper limit of normal reference values used in this study for each nail toxic metal concentration. **Table S3.** Associations between mixed metal concentrations and childhood nephrotic syndrome outcomes, by weighted quantile sum regression analysis. **Table S4.** Adjusted associations between toxic metal concentrations and childhood nephrotic syndrome outcomes. STROBE checklist.

## Footnotes

Supplementary File (PDF)

**Supplementary File.**
**Figure S1.** Nail toxic metal concentrations among 298 children with nephrotic syndrome. **Table S1.** Internal laboratory quality control measures of nail toxic metal concentration analysis. **Table S2.** Upper limit of normal reference values used in this study for each nail toxic metal concentration. **Table S3.** Associations between mixed metal concentrations and childhood nephrotic syndrome outcomes, by weighted quantile sum regression analysis. **Table S4.** Adjusted associations between toxic metal concentrations and childhood nephrotic syndrome outcomes. STROBE checklist.
