# Supplementary material for: Associations Between Toxic Metal Exposure and Childhood Nephrotic Syndrome
Source: Kidney Int Rep. 2026 Feb 12;11(4):106357. doi: 10.1016/j.ekir.2026.106357 (PMC12994064; doi:10.1016/j.ekir.2026.106357)
Supplement: Supplementary File (PDF) — Supplementary File. Figure S1. Nail toxic metal concentrations among 298 children with nephrotic syndrome. Table S1. Internal laboratory quality control measures of nail toxic metal concentration analysis. Table S2. Upper limit of normal reference values used in this study for each nail toxic metal concentration. Table S3. Associations between mixed metal concentrations and childhood nephrotic syndrome outcomes, by weighted quantile sum regression analysis. Table S4. Adjusted associations between toxic metal concentrations and childhood nephrotic syndrome outcomes. STROBE checklist. [file mmc1.pdf]

Supplementary Material

Table S1. Internal laboratory quality control measures of nail toxic metal concentration analysis

Analytic method	Quality control measures
**Mercury – TM.0813 (DMA-80 EVO) Solids**	1. R^2: > 0.9950<br>2. IPR (low level) avg: 95%<br>3. IPR (high level) avg: 98%<br>4. RPD in Sample Duplicate avg: N/A<br>5. Recovery CRM (IAEA-086) avg: 90%<br>6. Recovery MS & MSD avg: 101% & 101%<br>7. RPD in Spike Duplicate avg: 1%<br>8. MDL Abs: 0.0696 ng<br>9. MRL Abs: 0.2087 ng
**Other toxic metals (copper, arsenic, selenium, cadmium, tin, lead) – TM.0822 ICP-MS (Agilent 7700x ICP-MS) Solids**	1. R^2: > 0.9980<br>2. Recovery QCS/LFB: within 15% of the expected value<br>3. Recovery MS & MSD: within 30% of the known value<br>4. RPD in Sample Duplicates: within 30% control limit (or within 20% control limit for values greater than 100 x MDL)<br>5. RPD in MS & MSD: within 30% control limit<br>6. LRB: < 2.2 x MDL (or < 10% of lowest sample concentration, whichever is higher)<br>7. MB: < 2.2 x MDL (or < 10% of lowest sample concentration, whichever is higher)
R^2: Coefficient of determination, QCS: Quality control sample, CRM: Certified reference material, MS: Matrix spike, MSD: Matrix spike duplicate, RPD: Relative percentage difference, IPR & OPR: Initial & on-going precision and recovery, LFB: Laboratory fortified blank, LRB: Laboratory reagent blank, MDL: Method detection limit, MRL: Method reporting limit, MB: Method blank, SD: Standard deviation.	

Table S2. Upper limit of normal reference values used in this study for each nail toxic metal concentration

Toxic metal	Upper limit of normal (mcg/g)	Lower toxicity limit (mcg/g)	References
**Mercury**	2	2.5-10	S1-2
**Copper** [1]	20	Unknown, up to 59.2 in healthy controls	S3-6
**Arsenic**	1	1.8	S5, S7-8
**Selenium** [1]	3	Unknown, up to 7.1 in healthy controls	S5-6
**Cadmium** [1]	0.2	Unknown, up to 2.4 in health controls	S3, S5
**Tin** [1]	3.8	Unknown, up to 10 in healthy controls	S5
**Lead**	4	10-25	S8-10

[1] Lack of standardized reference values for upper limit of normal and lower toxicity limits of nail metal concentrations

Table S3. Associations between mixed metal concentrations and childhood nephrotic syndrome outcomes, by weighted quantile sum regression analysis

Outcome	Estimate for positive WQS (95% CI)	Estimate for negative WQS (95% CI)
Relapse rate	Beta -0.09 (-0.39, +0.21)	Beta +0.08 (-0.20, +0.37)
SRNS	NR [1]	NR [1]
FR or SDNS	OR 1.18 (0.58-2.39)	OR 0.82 (0.41-1.63)
Steroid-sparing medication use	OR 1.04 (0.66-1.65)	OR 1.03 (0.64-1.65)

WQS: weighted quantile sum, 95% CI: 95% confidence interval, SRNS: steroid resistant nephrotic syndrome, FR or SDNS: frequently relapsing or steroid dependent nephrotic syndrome, NR: not reported, OR: odds ratio

[1] Not reported due to small number of events (n=17) resulting in unstable estimates with very wide confidence intervals

Table S4. Adjusted associations between toxic metal concentrations and childhood nephrotic syndrome outcomes

Exposure [1]	Relapse rate during follow-up [2]	Initial SRNS	FR- or SDNS by 1-year after diagnosis	Steroid-sparing medication use during follow-up
	aRR (95%CI) [3]	aOR (95%CI) [3]	aOR (95%CI) [3]	aOR (95%CI) [3]
Log mercury concentration (mcg/g)	1.05 (0.95-1.15)	0.86 (0.54-1.30)	1.05 (0.85-1.30)	1.14 (0.93-1.41)
Log copper concentration (mcg/g)	1.18 (0.84-1.64)	NR [4]	0.83 (0.35-1.87)	1.30 (0.59-2.90)
Log arsenic concentration (mcg/g)	0.87 (0.67-1.12)	0.48 (0.05-2.47)	0.78 (0.43-1.41)	0.62 (0.33-1.12)
Log selenium concentration (mcg/g)	0.98 (0.65-1.47)	0.58 (0.02-9.30)	0.87 (0.34-2.21)	0.59 (0.22-1.51)
Log cadmium concentration (mcg/g)	0.89 (0.70-1.13)	0.50 (0.05-2.51)	0.80 (0.46-1.38)	0.65 (0.36-1.12)
Log tin concentration (mcg/g)	0.90 (0.69-1.16)	0.41 (0.04-2.19)	0.70 (0.38-1.26)	0.67 (0.36-1.21)
Log lead concentration (mcg/g)	0.88 (0.69-1.13)	0.47 (0.05-2.36)	0.56 (0.30-1.00)	0.65 (0.35-1.15)

All p-values >0.05. SRNS: steroid-resistant nephrotic syndrome, FR- or SDNS: frequently-relapsing or steroid-dependent nephrotic syndrome, RR: relative rate, OR: odds ratio, 95% CI: 95% confidence interval

[1] Toxic metal concentrations were log-transformed. All metal concentrations are reported in mcg/g of nail sample
[2] Relapse rate calculated as the total number of relapses per year of follow-up time
[3] Adjusted relative rates were calculated using negative binomial regression models, to account for over-dispersion. Adjusted odds ratios were calculated using logistic regression models. All models were adjusted for age at diagnosis, sex, and ethnicity
[4] Not reported (NR) due to low event rate leading to an unstable estimate and confidence interval

Figure S1. Nail toxic metal concentrations among 298 children with nephrotic syndrome

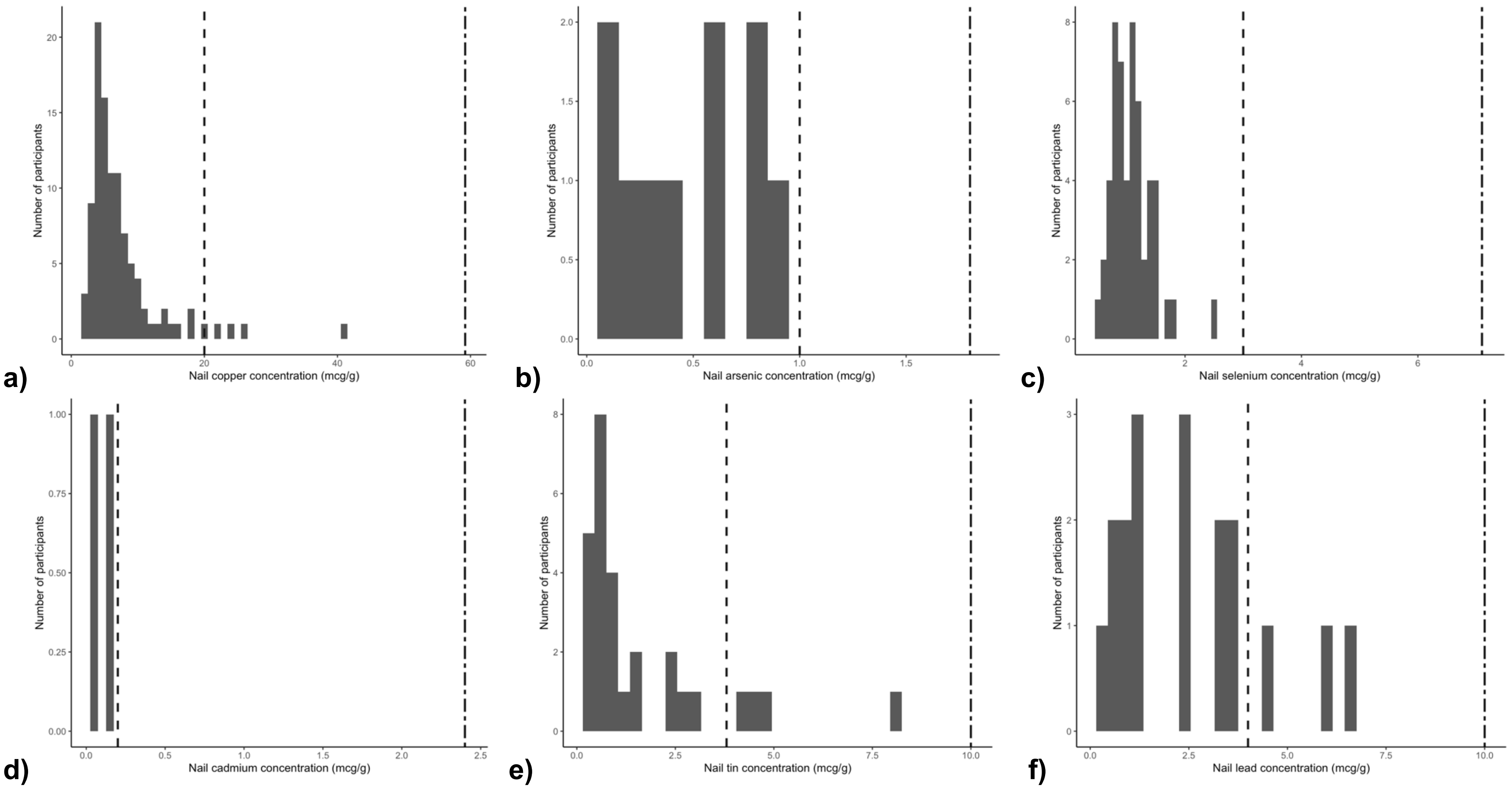

Supplement Figure 1 shows the distribution of participant nail toxic metal concentrations in mcg/g: a) copper, b) arsenic, c) selenium, d) cadmium, e) tin, and f) lead. In each figure, the single dashed line represents the upper limit of normal and the double dashed line represents the lower toxicity limit (in mcg/g).

The Strengthening the Reporting of Observational Studies in Epidemiology (STROBE) checklist

	Item No	Recommendation	Location in the manuscript where the item is reported
**Title and abstract**	1	(*a*) Indicate the study's design with a commonly used term in the title or the abstract	Title page, pg. 1
		(*b*) Provide in the abstract an informative and balanced summary of what was done and what was found	Abstract, pg. 2
**Introduction**			
Background/rationale	2	Explain the scientific background and rationale for the investigation being reported	Pg. 3
Objectives	3	State specific objectives, including any prespecified hypotheses	Pg. 4
**Methods**			
Study design	4	Present key elements of study design early in the paper	Pg. 4-5
Setting	5	Describe the setting, locations, and relevant dates, including periods of recruitment, exposure, follow-up, and data collection	Pg. 4-5
Participants	6	(*a*) Give the eligibility criteria, and the sources and methods of selection of participants. Describe methods of follow-up	Pg. 4-5
		(*b*) For matched studies, give matching criteria and number of exposed and unexposed	N/A
Variables	7	Clearly define all outcomes, exposures, predictors, potential confounders, and effect modifiers. Give diagnostic criteria, if applicable	Pg. 6-7
Data sources/ measurement	8*	For each variable of interest, give sources of data and details of methods of assessment (measurement). Describe comparability of assessment methods if there is more than one group	Pg. 4-6
Bias	9	Describe any efforts to address potential sources of bias	Pg. 6-8
Study size	10	Explain how the study size was arrived at	Pg. 4-5
Quantitative variables	11	Explain how quantitative variables were handled in the analyses. If applicable, describe which groupings were chosen and why	Pg. 6-7

Statistical methods	12	(*a*) Describe all statistical methods, including those used to control for confounding	Pg. 7-8
		(*b*) Describe any methods used to examine subgroups and interactions	Pg. 8
		(*c*) Explain how missing data were addressed	Pg. 7
		(*d*) If applicable, explain how loss to follow-up was addressed	Pg. 4-5
		(*e*) Describe any sensitivity analyses	N/A
**Results**			
Participants	13*	(a) Report numbers of individuals at each stage of study—eg numbers potentially eligible, examined for eligibility, confirmed eligible, included in the study, completing follow-up, and analysed	Pg. 9
		(b) Give reasons for non-participation at each stage	Pg. 9
		(c) Consider use of a flow diagram	N/A
Descriptive data	14*	(a) Give characteristics of study participants (eg demographic, clinical, social) and information on exposures and potential confounders	Pg. 9<br>Table 1
		(b) Indicate number of participants with missing data for each variable of interest	Table 1
		(c) Summarise follow-up time (eg, average and total amount)	Pg. 9
Outcome data	15*	Report numbers of outcome events or summary measures over time	Pg. 9<br>Table 2
Main results	16	(*a*) Give unadjusted estimates and, if applicable, confounder-adjusted estimates and their precision (eg, 95% confidence interval). Make clear which confounders were adjusted for and why they were included	Pg. 9-10<br>Table 3<br>Figure 1-3
		(*b*) Report category boundaries when continuous variables were categorized	Table 1
		(*c*) If relevant, consider translating estimates of relative risk into absolute risk for a meaningful time period	N/A
Other analyses	17	Report other analyses done—eg analyses of subgroups and interactions, and sensitivity analyses	Pg. 10-11
**Discussion**			
Key results	18	Summarise key results with reference to study objectives	Pg. 11
Limitations	19	Discuss limitations of the study, taking into account sources of potential bias or imprecision. Discuss both direction and magnitude of any potential bias	Pg. 14-15

Interpretation	20	Give a cautious overall interpretation of results considering objectives, limitations, multiplicity of analyses, results from similar studies, and other relevant evidence	Pg. 11-16
Generalisability	21	Discuss the generalisability (external validity) of the study results	Pg. 14-15
**Other information**			
Funding	22	Give the source of funding and the role of the funders for the present study and, if applicable, for the original study on which the present article is based	Pg. 17

Supplementary References

S1. World Health Organization: Guidance for identifying populations at risk from mercury exposure. Geneva, Switzerland: World Health Organization: World Health Organization;

S2. National Research Council (U.S.), editor: Toxicological effects of methylmercury. Washington, DC: National Academy Press;

S3. Ayyub A, Ahmad MuhammadS, Ikram N, Shan U-A: COPPER AND CADMIUM LEVELS IN HAIRS & NAILS;: An indicator of heavy metal pollution. Prof. Med. J. 20: 296–300, 2013

S4. Wilhelm M, Hafner D, Lombeck I, Ohnosorge FK: Monitoring of cadmium, copper, lead and zinc status in young children using toenails: comparison with scalp hair. Sci. Total Environ. 103: 199–207, 1991

S5. Blaurock-Busch E, Busch Y, Friedle A, Buerner H, Parkash C, Kaur A: Comparing the Metal Concentration in the Nails of Healthy and Cancer Patients Living in the Malwa Region of Punjab, India with a Random European Group – A Follow up Study. Br. J. Med. Med. Res. 5: 480–498, 2015

S6. Gutiérrez-González E, García-Esquinas E, de Larrea-Baz NF, Salcedo-Bellido I, Navas-Acien A, Lope V, et al.: Toenails as biomarker of exposure to essential trace metals: A review. Environ. Res. 179: 108787, 2019

S7. Hindmarsh JT, McCurdy RF: Clinical and environmental aspects of arsenic toxicity. Crit. Rev. Clin. Lab. Sci. 23: 315–347, 1986

S8. Rifai N, Chiu RWK, Young I, Burnham C-AD, Wittwer C, Tietz NW, editors: Tietz textbook of Laboratory Medicine. Seventh edition. St. Louis, Missouri: Elsevier;

S9. Dobbs MR, editor: Clinical neurotoxicology: syndromes, substances, environments. Philadelphia, PA: Saunders/Elsevier;

S10. Barbosa F, Tanus-Santos JE, Gerlach RF, Parsons PJ: A critical review of biomarkers used for monitoring human exposure to lead: advantages, limitations, and future needs. Environ. Health Perspect. 113: 1669–1674, 2005
